# Imaging and quantifying non-radiative losses at 23% efficient inverted perovskite solar cells interfaces

**DOI:** 10.1038/s41467-022-30426-0

**Published:** 2022-05-23

**Authors:** Stefania Cacovich, Guillaume Vidon, Matteo Degani, Marie Legrand, Laxman Gouda, Jean-Baptiste Puel, Yana Vaynzof, Jean-François Guillemoles, Daniel Ory, Giulia Grancini

**Affiliations:** 1 CNRS, École Polytechnique, IPVF, UMR 9006, 18, Boulevard Thomas Gobert, 91120 Palaiseau, France; 2 Institut Photovoltaïque d’Ile-de-France (IPVF), 18 Boulevard Thomas Gobert, 91120 Palaiseau, France; 3 Department of Chemistry and INSTM, University of Pavia, Via T. Taramelli 14, 27100 Pavia, Italy; 4 Électricité de France (EDF), R&D, 18 Boulevard Thomas Gobert, 91120 Palaiseau, France; 5 Dresden Integrated Center for Applied Physics and Photonic Materials (IAPP) and Center for Advancing Electronics Dresden (cfaed), Technische Universität Dresden, 01062 Dresden, Germany

**Keywords:** Solar cells, Solar energy and photovoltaic technology

## Abstract

Interface engineering through passivating agents, in the form of organic molecules, is a powerful strategy to enhance the performance of perovskite solar cells. Despite its pivotal function in the development of a rational device optimization, the actual role played by the incorporation of interfacial modifications and the interface physics therein remains poorly understood. Here, we investigate the interface and device physics, quantifying charge recombination and charge losses in state-of-the-art inverted solar cells with power conversion efficiency beyond 23% - among the highest reported so far - by using multidimensional photoluminescence imaging. By doing that we extract physical parameters such as quasi-Fermi level splitting (QFLS) and Urbach energy enabling us to assess that the main passivation mechanism affects the perovskite/PCBM ([6,6]-phenyl-C_61_-butyric acid methyl ester) interface rather than surface defects. In this work, by linking optical, electrical measurements and modelling we highlight the benefits of organic passivation, made in this case by phenylethylammonium (PEAI) based cations, in maximising all the photovoltaic figures of merit.

## Abstract (web summary)

In this work, charge recombination and losses in inverted solar cells with dual organic cations interfacial passivation are quantified by mapping physical parameters obtained via continuous wave and time resolved photoluminescence imaging techniques.

## Introduction

Inverted perovskite solar cells following a *p-i-n* structure have recently attracted enormous interest due to their enhanced stability and reduced hysteresis compared to the traditional *n-i-p* architecture^1,2^. However, *n-i-p* devices, where the perovskite absorber is deposited onto an electron transport layer, still result in higher power conversion efficiencies (PCE) reaching values up to 25.6%^3^ mainly due to a better carrier extraction. One of the most common strategies employed to limit non-radiative losses and therefore improve perovskite solar cells (PSC)s efficiency relies on the use of interface passivation agents such as low dimensional halide perovskites^2,4,5^, organic cations^6,7^ or self-assembled monolayers (SAM)^8,9^. In our recent work, we have developed an innovative strategy for the optimisation of *p-i-n* state-of-the-art inverted solar cells^10^, consisting of a dual passivation method at both the top and bottom interfaces of the perovskite active layer by introducing large organic cations at these interfaces. This resulted in improved performances with PCE beyond 23%, among the highest reported so far^10^. Specifically, two large organic A- site cations, i.e. 4-chloro-phenylethylammonium iodide (Cl-PEAI) and 4-fluoro-phenylethylammonium iodide (F-PEAI), were introduced at the interfaces of the perovskite absorber with both hole transport layer (HTL) and electron transport layer (ETL). Despite the clear improvement, questions regarding the physics behind the modified interfaces are still open^11^. Issues regarding the completeness of the coverage of the passivating layer^12^, whether a 2D perovskite is formed or not^13,14^, and the possible effects on carrier extraction remain not fully understood. In a more general way, several studies focused on the recombination dynamics at the perovskite/selective interfaces by tracking the photoluminescence signal and its intensity^15–17^. For instance, the contributions of bulk and interfacial recombination currents were decoupled through the measurements of the quasi-Fermi level splitting (QFLS) of the individual layer by all-optical techniques^18^. Stolterfoht and co-workers used transient and absolute photoluminescence (PL) imaging to visualise non-radiative recombination pathways at modified perovskite/C_60_^19^ electron transport layer. Here, we combine steady-state and time-resolved multidimensional photoluminescence imaging techniques to probe the main optoelectronic and transport properties of the optimised *p-i-n* devices. First, our results demonstrate that the Cl-PEAI and F-PEAI cations deposition result in a homogenous coverage of the perovskite surface. Second, we verify that no layered perovskite is formed. Moreover, we identify that interfacial passivation is the main mechanism driving the improvement of the device open circuit voltage (V_oc_), as the boost of QFLS and the reduction of surface recombination rate are observed only after PCBM (ETL) deposition. Instead, the introduction of the cations at the perovskite bottom interface (HTL/perovskite) helps in the perovskite crystallisation process but does not significantly reduce the losses at that specific device interface, as demonstrated by the quantitative analysis of the QFLS.

## Results

### Dual interfacial modified devices

**Fig. 1:**
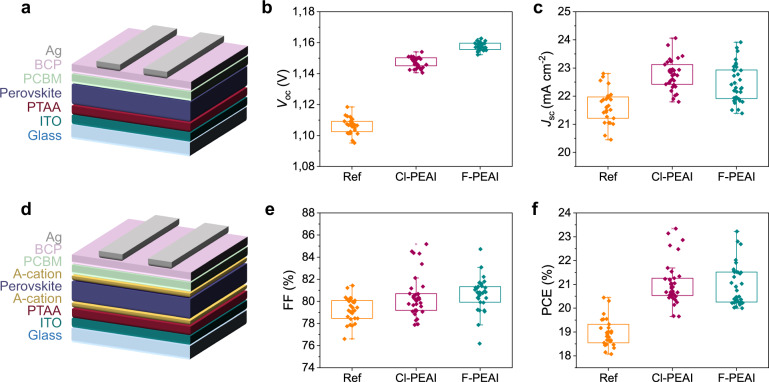
Electrical characterisation of the samples. **a** Schematic of the reference samples **d** Schematic of the A-cation *p-i-n* devices with dual interfacial modification. Photovoltaic characteristics of reference (orange), Cl-PEAI (purple) and F-PEAI (teal blue) devices. **b** Open circuit voltage (*V*_oc_). **c** Short circuit current (*J*_sc_). **e** Fill Factor (FF). **f** Power conversion Efficiency (PCE).

The devices under investigation follow a *p-i-n* structure, as shown in Fig. 1a, d. The full stack includes: glass/Indium Tin Oxide (ITO)/Poly[bis(4-phenyl)(2,4,6-trimethylphenyl)amine (PTAA)/A-cation/perovskite/A-cation/[6,6]-phenyl-C_61_-butyric acid methyl ester (PCBM)/Bathocuproine (BCP)/silver. Ionic salts, namely piperidinium salt [BMP]^+^[BF_4_]^−^^20^, were added to the mixed cation double halide composition (Cs_0.05_(FA_5/6_MA_1/6_)_0.95_Pb(I_0.9_Br_0.1_)_3_). Three different types of cells were fabricated: a reference cell (Fig. 1a) and devices with dual interfacial modification by F-PEAI and Cl-PEAI cations (Fig. 1d). The cations, commonly used as precursor for 2D perovskite formation, were added in very low concentrations (of 0.5 mM) at the top interface, meanwhile 20 mM were added at the bottom interface. The use of such low concentrations did not lead to the formation of a 2D Ruddlesden-Popper (RP) phase^10^, as confirmed by XRD analysis reported in Supplementary Fig. S1, showing the characteristic perovskite reference peaks. However, as recently reported by Gharibzadeh et al^21^, 2D organic cations can be also employed to passivate both grain boundaries and interfacial defects in *p-i-n* configuration devices, allowing to achieve a substantial enhancement of the device performances. In this case the use of the top and bottom modification with the large organic cations is meant to improve the interfaces between the perovskite and the selective charge transport layers by simultaneously reducing the defect density and therefore the non-radiative recombination.

The main photovoltaic characteristic parameters for the reference cell and the A-cations devices are reported in Fig. 1b, c, e and f. The addition of the cations induced an increase of all the main photovoltaic characteristics. The PCE of the devices in which both absorber interfaces were modified by A-cations is clearly enhanced, exhibiting values up to 23.34% for the Cl-PEAI and 23.22% for the F-PEAI. In particular, we can note a clear increase of the *V*_oc_ from 1.10 V for the reference to 1.15 V using Cl-PEAI and exceeding 1.16 V using F-PEAI, which corresponds to a tenfold decrease of the dark current, i.e. of the carrier recombination rate. The current also slightly improved, in contrast with some previous observations in the literature, where the full formation of a 2D perovskite layer was acting as a blocking layer^22^. Remarkably, fill factor reached values up to 85 % on the Cl-PEAI and F-PEAI devices, within the highest ever reported for halide perovskite solar cells. Also, the devices do not show any hysteretic behaviour, as is shown in Supplementary Fig. 5.

### Quantification of non-radiative losses at the interfaces

**Fig. 2:**
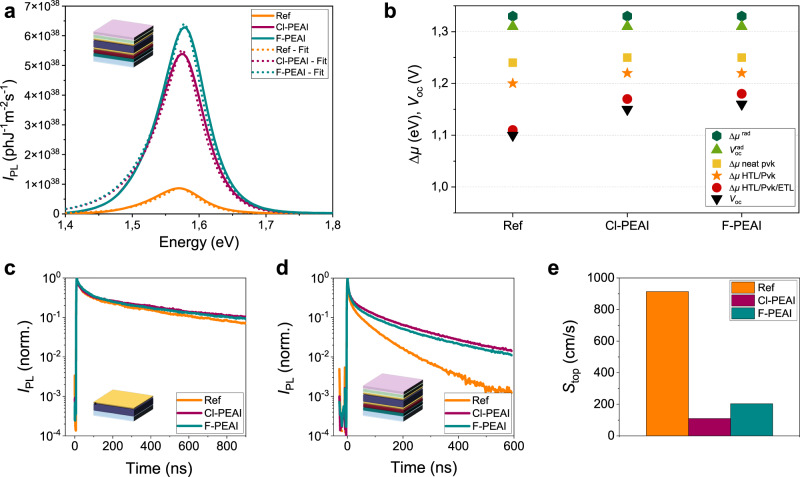
Spatially averaged photoluminescence analysis in the continuous wave and time-resolved regime. **a** Photoluminescence average spectra and corresponding fits acquired on the stack glass/ITO/PTAA/perovskite/PCBM/BCP samples. PL spectra were acquired on reference (orange), Cl-PEAI (purple) and F-PEAI samples (teal blue). **b** QFLS values extracted from PL spectra for neat perovskite, half cells and full devices compared with radiative QFLS *Δµ*^rad^, open circuit voltage *V*_oc_ and radiative open circuit voltage *V*_oc_^rad^. TR-FLIM (spatially integrated) decays acquired at 10^11 ^ph.cm^−2^ fluence for **c** perovskite layers deposited on glass, top illumination and **d** full cells without top bottom electrode, top illumination. **e** Comparison of the fitted top surface recombination rate on full devices.

To better understand the origin of this evident improvement in terms of device performances we employed multi-dimensional photoluminescence and analysed the datasets by employing physical models to quantify the key parameters governing the carrier recombination and transport. Specifically, we used two different set-ups to investigate both steady-state and transient regimes, namely a Hyperspectral Imager (HI)^23^ and a Time Resolved Fluorescence Imaging (TR-FLIM)^24^ set-up. Photoluminescence (PL) spectrum images were acquired by using a blue laser (405 nm) and illuminating the samples from the thin films side to avoid optical artefacts induced by the glass. We acquired a series of PL calibrated maps of neat perovskite thin films deposited on glass, half devices (glass/ITO/PTAA/perovskite) and full devices without the silver top electrode (glass/ITO/PTAA/perovskite/PCBM/BCP) of reference and Cl-PEAI or F-PEAI samples. Notably, the A-cations samples do not exhibit any 2D perovskite emission peak, as shown in Supplementary Fig. 3. The lack of a 2D perovskite formation is thus further confirmed, corroborating the XRD measurements. The spatially averaged and absolutely calibrated values of each map were fitted by using a model based on Planck’s law^25,26^ (see Fig. 2a for PL spectra and fits of full devices), allowing to determine the QFLS values for each set of samples, reported in Fig. 2b and in Table 1. In Table 1 we also report the radiative quasi–Fermi level splitting (*Δµ*^rad^) and the radiative open circuit voltage (*V*_oc_^rad^). The *Δµ*^rad^ is defined as:

1 $${\triangle {\mu }}^{{{{{{\rm{rad}}}}}}}={kT}\cdot {{{{{\rm{ln}}}}}}\left(\tfrac{{I}_{{{{{{\rm{ph}}}}}}}}{{q\phi }_{{{{{{\rm{em}}}}}}}}\right)$$

where *I*_ph_ is the photocurrent due to the considered illumination and *kT* the thermal energy of the charge carriers and *q* is the elementary charge. The term ${\phi }_{{{{{{{\mathrm{em}}}}}}}}$ is the PL emission of the absorber in thermal equilibrium with its surrounding at 300 K. It means that only the thermal photons emitted in the dark by the surrounding excite the device, which in return emits the very minimum PL photons flux possible. This PL flux depends on the bang gap energy, the temperature and the absorption coefficient, which is affected by the defect density.

**Table 1:** Values of radiative QFLS *Δµ*^rad^; QFLS *Δµ* for the different stacks (neat perovskite (pvk), half cells (glass/ITO/PTAA/pvk) and full stacks (glass/ITO/PTAA/pvk/PCBM/BCP)); *V*_oc_^rad^ and *V*_oc_ for reference, Cl-PEAI and F-PEAI samples.

Sample	*Δµ*^rad^ (eV)	*Δµ* pvk (eV)	*Δµ *HTL/pvk (eV)	*Δµ *HTL/pvk/ETL (eV)	*V*_oc_^rad^ (V)	*V*_oc_ (V)
Reference	1.33	1.24	1.20	1.11	1.31	1.10
Cl-PEAI	1.33	1.25	1.22	1.17	1.31	1.15
F-PEAI	1.33	1.25	1.22	1.18	1.31	1.16

Moreover, we defined the radiative open circuit voltage *V*_oc_^rad^ as:

2 $${V}_{{{{{{\rm{oc}}}}}}}^{{{{{{\rm{rad}}}}}}}=\frac{{{{{{{\mathrm{kT}}}}}}}}{q}\cdot{{{{{\rm{ln}}}}}}\left(\frac{{J}_{{sc}}}{{J}_{0{{{{{\rm{rad}}}}}}}}\right)$$

Where kT is the thermal energy of the charge carriers, *q* is the elementary charge, *J*_sc_ is the short-circuit current density of the solar cell and *J*_0rad_ is the radiative current^27,28^. Specifically:

3 $${J}_{0{{{{{\rm{rad}}}}}}}=q\int {{{{{\rm{EQE}}}}}}(E){\phi }_{{{{{{\rm{BB}}}}}}}^{300{{{{{\rm{K}}}}}}}\left(E\right){dE}$$

with EQE being the external quantum efficiency of the full devices.

We considered the radiative QFLS (*Δµ*^rad^) as the radiative limit for the optical measurements and the *V*_oc_^rad^ as the upper limit for the open-circuit voltage measured by electrical characterisation. The small difference of the values determined for the two limits arises from the fact that the *V*_oc_^rad^ depends not only from optical properties of the absorber and the radiative recombination but also from the collection and the injection of carriers.

We obtained values of QFLS for neat perovskite thin films equal to 1.24 eV for the reference sample and 1.25 eV for the two A-cations samples, corresponding to 80–90 meV losses if compared to the radiative QFLS (*Δµ*^rad^) that is equal to 1.33 eV in all cases. Interestingly, the main impact of the organic cation passivation is observed for full devices. In this case, the difference between reference and A-cations containing samples is in the range of 60–70 meV, proving that the cations have drastically reduced the non-radiative recombination at the interface absorber/ETL. Conversely, when considering neat perovskite or half devices the addition of the cations resulted in an improvement of only 10–20 meV. Therefore, the main passivation mechanism acting relates to the interfacial passivation at the interface absorber/ ETL. The introduction of the organic cations at the interface HTL/absorber favoured the perovskite crystallisation on the PTAA surface, but did not drastically reduce the losses at that interface. It is worth noting that the interface perovskite/PCBM appears to be particularly critical for device optimisation. The fast recombination observed at this specific interface represents a major loss mechanism for solar cell operation^29^. Moreover, we linked the QFLS of full devices to the *V*_oc_ measured on full devices^30^ by finding a good agreement between the two values, thus confirming the direct relationship between optical and electrical measurements. A similar approach to evaluate the recombination processes of 3D-2D interfaces in a *n-i-p* architecture was performed by Sutanto et al.^22^, where the interfacial passivation was considered as the main process affecting the losses. However, cation interfacial passivation, not only reduce the non-radiative recombination, but also favour the carrier extraction that, on the contrary, is limited in the case of full 2D perovskite formation at the interfaces, acting as a barrier for the photo-generated carriers^22^. Finally, we determine the total losses due to non-radiative recombination in full devices by comparing the radiative voltage radiative voltage *V*_oc_^rad^ with the measured *V*_oc_ (ΔV= *V*_oc_^rad^ −*V*_oc_). In the reference sample Δ*V*_ref_ is equal to 0.21 eV, while in the A-cations devices ΔV corresponds to 0.16 eV in the case of Cl-PEAI and to 0.15 eV in the case of F-PEAI, thus confirming the beneficial role played by the addition of the cations on the charge losses.

To gain further insights regarding the carrier dynamics, we performed TR-FLIM analysis on two sets of samples: (i) thin films on glass and (ii) full cell devices (without the Ag metallic layer to allow for illumination from both sides). For both sets of samples, we analysed a reference sample with only bulk perovskite as well as Cl-PEAI and F-PEAI samples. The experiments were performed using a wide field illumination via a pulsed 532 nm laser with repetition rate of 40 kHz. For each sample, the fluence of the laser was varied from low (~10^11^ ph.cm^−2^) to high level (~1.5 × 10^12^ ph.cm^−2^) and all the samples were measured with both top and bottom illumination (and light collection). A complete list of the available data and conditions is reported in the Supplementary Information.

Decays averaged over the image for low fluence (~10^11^ ph.cm^−2^) by illuminating the samples from the thin films side are presented in Fig. 2c (neat perovskite) and **2d** (full devices). In the case of bare absorbers, the three decays are very similar, whereas for the full devices, the samples with the added A-cation layers show slower decay than the reference. We estimate that the slightly higher PL level obtained for the Cl-PEAI bare absorbers is not discriminating enough to conclude that this cation has a better passivation effect than its F-PEAI counterpart. Slower decays of multilayer samples do not necessarily imply better devices as passivation layers could hinder the amplitude of the current of charges flowing out of the cell. In this case, considering also that the short-circuit currents slightly increased upon modification with the A-cations (see Fig. 1c), we can conclude that this passivation approach allows to reduce non-radiative recombination at the perovskite-charge transport layer interfaces while allowing for high currents to flow. Transient measurements thus confirmed that the passivation plays a key role only at the perovskite-charge transport layer interface, as also observed in the steady-state study. Importantly, the time-resolved analysis also proves that high non-radiative recombination is responsible for the fast decay of the reference cell of Fig. 2d, that therefore cannot be attributed to carrier extraction as sometimes reported in the literature^31,32^.

Next, we treated the datasets by using drift-diffusion model in order to quantify the induced passivation. However, fitting decays of multilayer samples remains challenging, as an incorrect modelling of the layer stack, of the band alignment or of the boundary conditions can easily lead to data misinterpretation^16,33^. To overcome this issue, we chose to fit the simplest model possible, that is based on a thin film without the neighbouring extracting layers. Supplementary Figure 10 represents the fitting results of the cells’ decays, the green dotted line is the fitted model, while the coloured curves correspond to the experimental data. We fit a unique drift-diffusion model to represent simultaneously four curves, i.e. decays acquired in bottom and top configuration at low and high fluence. The fitted parameters are the top and bottom surface recombination velocities – all the other parameters are fixed, see Supplementary Materials for more details. We consider the bulk non-radiative recombination to be negligible as compared to surface recombination and assign all non-radiative recombination to the surfaces. The fitting results for bottom surface recombination velocity on full devices are presented in Fig. 2d. We observe a drastic reduction of the recombination velocities at the top surface (*S*_top_) for the two passivation strategies. In particular, S_top_ was reduced by a factor varying from nine to five going from ~900 cm/s for the reference to ~110 cm/s and ~200 cm/s for the Cl-PEAI and F-PEAI samples respectively, as shown in Table 2. For unpassivated classical semiconductors, surface recombination velocities are of the order of 10^5^–10^6 ^cm/s^34–36^, while passivated surfaces may reach low values in the range of 10–10^2^ cm/s^34,37,38^. For perovskite films, even unpassivated, we^39,40^ and others^34,41^ have found surface recombination velocities of the order of 10^3^ cm/s which are fully compatible with our present analysis. Again, the TR-FLIM results are consistent with those obtained via the hyperspectral studies, showing that the passivation mainly takes place at the perovskite/ETL interface.

**Table 2:** Top and bottom fitted interface recombination velocity for reference and A-cations full cells.

Device (full cells)	*S*_top_ (cm/s)	*S*_bot_ (cm/s)
Reference	914 ± 160	389 ± 29
Cl-PEAI	110 ± 3	64 ± 2
F-PEAI	203 ± 6	39 ± 2

### Quantitative optical microscopy analysis

**Fig. 3:**
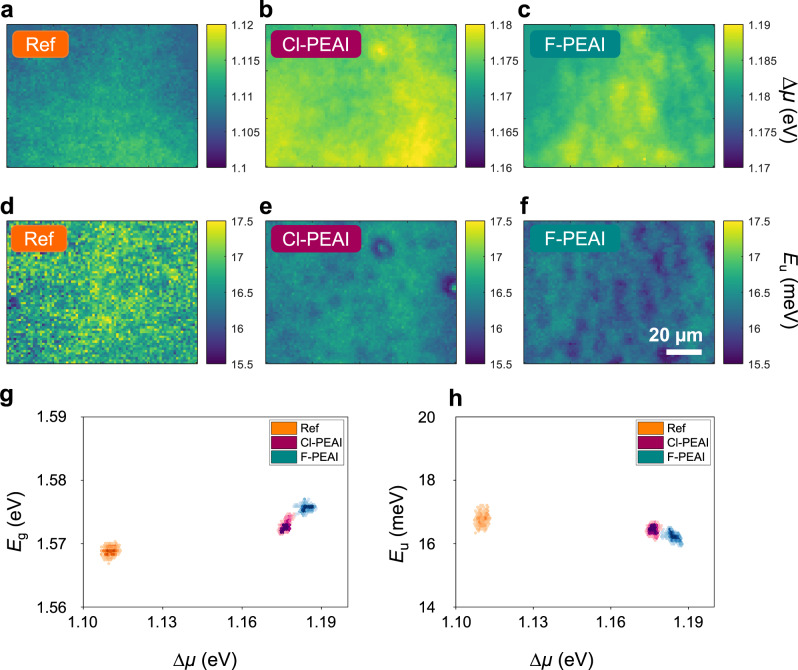
Continuous wave photoluminescence imaging analysis. Hyperspectral measurements on the stack glass/ITO/PTAA/perovskite/BCP/PCBM. quasi-Fermi level splitting (Δµ) maps for **a** reference sample, **b** Cl-PEAI and **c** F-PEAI samples. Urbach Energy maps for **d** reference sample, **e** Cl-PEAI and **f** F-PEAI samples. **g** Correlation between energy gap and QFLS. **h** correlation between Urbach Energy and QFLS. The scalebar applies to all images.

It is noteworthy that the passivation of the top surface is achieved by incorporating the A-cations into the antisolvent used for triggering the perovskite layer crystallisation, thus raising the question regarding the homogeneity of passivation obtained by this method. In order to better assess the homogeneity of the passivation over the samples surface, we mapped the optoelectronic properties by spectrally and temporally resolved photoluminescence imaging analysis on the full stack without the silver rear electrode. We first fitted the PL spectra pixel-by-pixel^39,42^ by using the model proposed by Katahara and Hillhouse^43,44^ to extract key physical parameters like the bandgap energy (*E*_g_), the quasi-Fermi levels splitting (Δμ or QFLS) and the Urbach energy (*E*_u_) from absolutely calibrated spectral images. More details on the fitting model are provided in the Supplementary Information section. The maps of the QFLS are shown in Fig. 3a–c. All the samples, reference and most importantly cation modified samples, show a good homogeneity with a standard deviation in the order of 0.006 eV. Moreover, no areas with values of QFLS comparable to pure 3D cells, i.e. in the range of 1.110 ± 0.013 eV, can be observed on the Cl-PEAI and F-PEAI samples, proving the uniformity of the passivation effect induced by the cations. The introduction of the large cations has thus produced a clear reduction of non-radiative losses and importantly we demonstrate that the passivating agents act uniformly on the whole surface of the solar cell. However, in the three cases, small spatial fluctuations in the order of few meV in the 10 µm range are distinguishable. These minor inhomogeneities may be due to the spin-coating process. In Fig. 3d–f we investigate the variations of the *E*_u_ which again exhibits a good homogeneity. The determined values are in line with *E*_u_ values reported in the literature and measured by different characterisation methods^45^.

To highlight the correlations between the different optoelectronic properties, as well as the statistic distribution of the determined parameters over the maps, we report the correlation between *E*_g_ and QFLS and between *E*_u_ and QFLS measured at each pixel, as shown in Fig. 3g, h. All the physical parameters, namely *E*_g_, QFLS and *E*_u_, have a small statistical dispersion. The introduction of the A-cations results in a minimal increase of the gap of the material in the order of few meV but significantly impacts the QFLS that shows a substantial raise of its average value from 1.11 eV up to 1.18 eV. A very small variation of *E*_u_ of ~1 meV is observed when comparing the reference to the A-cations passivated devices. Moreover, this analysis suggests that the addition of the cations does not significantly affect the bulk recombination kinetics of the absorber as QFLS (in the case of bare absorbers), *E*_g_ and the *E*_u_, do not vary significantly after the A-cations addition.

**Fig. 4:**
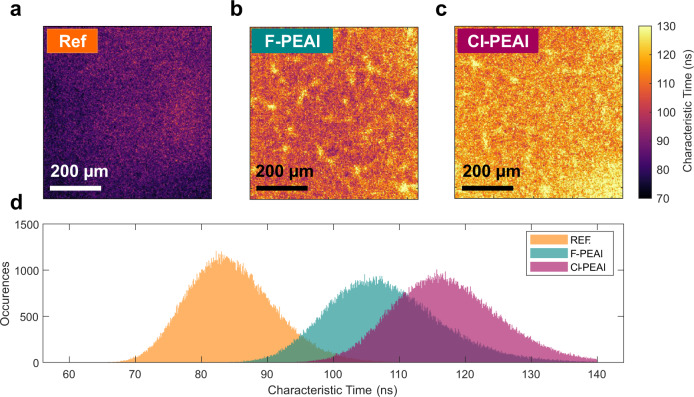
Time-resolved photoluminescence imaging analysis. **a**–**c** Map of decay time obtained on full-stacks (glass/ITO/PTAA/perovskite/BCP/PCBM) for the high fluence (1.5 × 10^12 ^ph.cm^−2^) acquisition. The homogeneity of the A-cations devices is comparable to the neat reference cell. **d** Histogram of the maps, showing the passivation effect of the layers.

Finally, we wanted to further confirm the homogeneity of the A-cation deposition as well as their role in the interface physics. To do so, we used TR-FLIM acquisitions to map the local decay times, often called “lifetime”, for the three full stacks (glass/ITO/PTAA/perovskite/BCP/PCBM), as shown in Fig. 4a–d. In general, the definition of “lifetime” is ambiguous, as it does not represent an intrinsic property of the material (e.g. it is dependent on the excitation conditions), and does not always correlate well with the electrical figures of merit, such as the open circuit voltage^15^. For these reasons we employ here the term decay time. If we place ourselves in the correct setting - after the carrier distribution has homogenised in depth and with low radiative recombination - the decay time of PL is a measure of the non-radiative recombination. In the case of triple cation mixed halide perovskite thin films this corresponds to a few tens to one hundred nanoseconds after the laser pulse^46^. We use then the decay times to map the non-radiative recombination in our devices. To get a high signal to noise ratio for the images we used the high fluence (1.5 × 10^12^ ph.cm^−2^) acquisitions, as reported in Fig. 4a–d. Here the decay time is defined as the inverse slope of the logarithm of the local decay fitted between 90 ns and 500 ns after the laser pulse, see SI for more details. Figure 4a–c are thus representative of the non-radiative recombination in the devices. Overall, an excellent homogeneity was achieved at the 1 mm^2^ scale for the F-PEAI and Cl-PEAI samples. The histograms of Fig. 4d display that the difference between reference and modified samples in terms of non-radiative recombination is statistically significant. The mean decay time on the maps extracted from the high fluence acquisitions are found to be 84.6 ns for the reference, 107.9 ns for the F-PEAI and 117.35 ns for Cl-PEAI. The images of the passivated layers show a slightly wider distribution of decay time, compared to the reference sample with a standard deviation of 6.7 ns for the reference and 8.7 ns and 8.2 ns for the F-PEAI and Cl-PEAI, respectively. This analysis provides us with an approximate estimate for the uncertainty and noise level on the images. The decay time maps thus show the homogeneity of the interfacial cation addition treatment resulting in a uniform improvement of decay times for the A-cation modified devices, further confirming the reduction of non-radiative recombination.

Moreover, we provide the decay times for the full stacks when the carrier density of the transient experiment is ~10^15^ cm^−3^, which is the order of magnitude of carrier density expected at 1 sun continuous illumination condition for perovskite devices close to the radiative limit^47^. To obtain these values of decay time, we used a procedure described in the SI for the low fluence (10^11^ ph.cm^−2^) acquisitions that yields decay times of 102 ns (±7%) for the reference cell, 254 ns ±(3%) for the Cl-PEAI cell and 261 ns (±3%) for the F-PEAI cell. For the low fluence dataset, the local signal to noise ratio of the maps is too low for the local determination of the decay time and therefore we do not show maps for this illumination condition.

## Discussion

In conclusion, we have investigated the interface recombination dynamics related to an emergent passivation approach in the field of perovskite solar cells based on the introduction of large cations at the absorber interfaces by coupling electrical and optoelectronic characterisation methods and modelling. In particular, we demonstrated that the introduction of large organic cations such as Cl-PEAI and F-PEAI resulted in a drastic reduction of interfacial recombination processes leading to PCE up to 23.34%, one of the highest ever reported for a* p-i-n* architecture. Importantly, we proved that this passivation approach does not significantly act on the surface defects of the absorber layer but drastically reduce the non-radiative recombination at the interface perovskite/PCBM. Indeed, the major improvement in terms of QFLS was observed after the deposition of the ETL, with an increment from 1.11 eV to 1.18 eV for passivated devices. Conversely, in the case of neat perovskite thin films the QFLS remained almost constant after the two cations deposition. The same behaviour was also observed for transient measurements that showed identical decays in the three cases. Finally, photoluminescence maps and decay times maps of full devices proved that the cations were uniformly deposited over the perovskite surface at the micrometre scale and that the main bulk optoelectronic properties of the absorber such as the energy gap and the Urbach energy were homogenous at a local level. This work provides insights into the processes occurring at the interfaces of highly efficient perovskite solar cells, which are fundamental for further progress in the field and makes this passivation method extremely promising to approach the theoretical limit of perovskite devices.

## Methods

### Materials

Unless otherwise stated, all materials were purchased from Sigma-Aldrich or Alfa Aesar and used as received. Perovskite films and devices were fabricated using PbI_2_ and PbBr_2_ (99.99% purity) purchased from TCI, organic halide salts purchased from GreatCell Solar and Cesium Iodide (99.99% purity) purchased from Alfa Aesar. The poly(triaryl amine) (PTAA) was purchased from Sigma-Aldrich. The PC61BM was purchase from Solenne. The bathocuproine (BCP; sublimed grade, 99.99% purity). All the anhydrous solvents were purchased from Acros Organics.

### Perovskite film preparation and device fabrication

Pre-patterned ITO/glass substrates were sequentially cleaned with acetone and isopropanol by ultrasonication for 15 min. The ITO/glass substrates were then dried with N2 and treated with oxygen plasma at 100 mW for 10 min. The HTL and the perovskite films were fabricated in a drybox (relative humidity <2 %), while the ETL and the contacts were deposited inside a glovebox filled with inert atmosphere N_2_. For reference devices, a hole transport layer of ∼10 nm thickness made of PTAA with a concentration of 1.5 mg ml^−1^ dissolved in toluene was spin-coated at a speed of 2000 rpm for 40 s and then annealed at 100 °C for 10 min. After the annealing step, the samples were washed by DMF by spin-coating it on the prepared PTAA films at 4000 rpm for 30 s. The perovskite precursor solution (1.2 M) composed of mixed cations (Pb, Cs, FA and MA) and halides (I and Br) was dissolved in mixed solvent (DMF/DMSO = 4/1) according to a formula of Cs_0.05_(FA_5/6_MA_1/6_)_0.95_Pb(I_0.9_Br_0.1_)_3_ with an excess of PbI_2_ of 1%. The piperidinium salt [BMP] + [BF4] − was dissolve in the perovskite solution obtained with the molar ratio 0.25 mol %. The perovskite is deposited via a two-step spin-coating procedure with 1000 r.p.m. for 12 s and 5000 r.p.m. for 27 s was adopted for the preparation of perovskite films. A mixture of antisolvents (CB/IPA = 9/1, 150 μl) was dripped on the spinning substrate during the 21 s of the second spin-coating step. Subsequently, the sample was annealed at 100 °C for 30 min. The electron transport layers were dynamically deposited from a PC61BM solution (20 mg/mL in CB) and spin-coated onto the perovskite layer at the speed of 2000 rpm for 30 s (with a ramping speed of 1000 rpm/s) and annealed for 10 min at 100 °C. Next, thin layers of BCP (0.5 mg/mL in IPA) were spin-coated at 4000 rpm for 30 s (with a ramping rate of 1000 rpm/s) as hole blocking layers. The devices with an area of 4.5 mm^2^ were completed by thermally evaporating of Ag (80 nm). The devices with modified interfaces were prepared by dissolving a small amount of the A-cations in DMF (20 mM) used for washing the PTAA and in the mixture CB/IPA (0.5 mM) used in the antisolvent step.

### Photovoltaic device characterisation

Current density-voltage measurements were performed in ambient conditions under simulated AM 1.5 light with an intensity of 100 mW cm^−2^ (Abet Sun 3000 Class AAA Solar Simulator). The intensity was calibrated using a Si reference cell (NIST traceable, VLSI), and corrected by measuring the spectral mismatch between the solar spectrum, reference cell, and the spectral response of the PV device. The mismatch factor obtained was approximately 1.1. Cells were scanned using a Keithley 2450 source measure unit from 1.2 to 0 V and back, with a step size of 0.025 V and a dwell time of 0.1 s, after light soaking for 2 s at 1.2 V. The pixel area was 3 mm × 1.5 mm.

### XRD

XRD patterns were measured in ambient air using a Bruker D2 Phaser diffractometer equipped with a Cu-Anode (*λ* =  1.54060 Å) and a LYNX-EYE detector in 1D mode. All scans (coupled 2θ/θ, 2*θ* = 10°–45°, step size 0.02°) were background corrected using the Bruker Diffrac.Eva software.

### Hyperspectral characterisation

The hyperspectral imaging system records a luminescence intensity signal along three dimensions {x,y,λ}. The set-up is composed by a home-built microscope with Thorlabs optomechanical elements, a 2D bandpass filtering system from company Photon Etc with 2 nm resolution, and a 1Mpix silicon-based CCD camera PCO1300. The sample was illuminated (*λ* = 405 nm) through an infinity-corrected ×50 Nikon objective with numerical aperture of 0.6, and the luminescence is collected through the same objective. The excitation beam and luminescence signals are separated with appropriate Thorlabs dichroic beam splitter (DMLP 495) and filters (FESH 450, FELH 450). The 2D luminescence signal is corrected for each pixel of the sensor from the spectral transmissions along all the optical path, from the read noise and dark current noise of the camera. The incident photon flux measured was 100 mW/cm^2^, corresponding to 1 sun equivalent photon flux. We obtained this photon flux by dividing the measured power of the LED used for the illumination under the objective over the LED spot size at the working distance of the objective. All the acquisitions were performed in nitrogen atmosphere. Post-treatment of the data cubes includes a deconvolution and fit to the generalised Planck law, which are realised with a dedicated Matlab routine employing the Levenberg−Marquardt algorithm.

### TR-FLIM characterisation

The TR-FLIM setup records luminescence intensity over an imaging sensor and as a function of time. We used a Princeton Instrument PiMAX4 gated camera. We used 3 ns wide temporal gates that we slid in time to record the local decays of the films. The illumination was performed with a Coherent Laser (*λ* = 532 nm, pulse width 15 ps), defocused and homogenised by a rotating diffuser to obtain a flat and homogenous wide field excitation. The repetition rate of the laser was set to 40 kHz.To estimate the fluences, the wide field illumination was imaged with a portable CCD array to obtain the illumination area while the incident power was also measured. A ×10 objective was used both for excitation and collection, and the laser was filtered out with a DMLP650R beam splitter as well as with a FEL0680 filter. Each acquisition was repeated two times in a row to check for any reproducibility issue (none was found) and to obtain a better average signal to noise ratio.

### Reporting summary

Further information on research design is available in the Nature Research Reporting Summary linked to this article.

## Supplementary information

Supplementary Information

Solar Cells Reporting Summary

## Data Availability

The data that support the findings of this study are available in the following repository: 10.6084/m9.figshare.19430450.

## Code availability

The codes that support the findings of this study are available from the corresponding authors upon reasonable request.
