# Supplementary material for: Imaging and quantifying non-radiative losses at 23% efficient inverted perovskite solar cells interfaces
Source: Nat Commun. 2022 May 23;13:2868. doi: 10.1038/s41467-022-30426-0 (PMC9126963; doi:10.1038/s41467-022-30426-0)
Supplement: Supplementary file 1 — Supplementary Information [file 41467_2022_30426_MOESM1_ESM.pdf]

Supplementary Information

Imaging and Quantifying non-radiative Losses at 23% Efficient Inverted Perovskite Solar Cells Interfaces

Stefania Cacovich[1*], Guillaume Vidon[2], Matteo Degani[3], Marie Legrand[2,4], Laxman Gouda[3], Jean-Baptiste Puel[2,4], Yana Vaynzof[5], Jean-François Guillemoles[1], Daniel Ory[2,4], Giulia Grancini[3*]

[1] CNRS, École Polytechnique, IPVF, UMR 9006, 18, Boulevard Thomas Gobert, 91120 Palaiseau, France

[2]IPVF, Institut Photovoltaïque d'Ile-de-France, 18 Boulevard Thomas Gobert, 91120 PALAISEAU, France

[3]Department of Chemistry and INSTM, University of Pavia, Via T. Taramelli 14, 27100 Pavia, Italy

[4]Électricité de France (EDF), R&D, 18 Boulevard Thomas Gobert, Palaiseau, 91120, France

[5] Dresden Integrated Center for Applied Physics and Photonic Materials (IAPP) and Center for Advancing Electronics Dresden (cfaed), Technische Universität Dresden, 01062 Dresden

Corresponding Author

emails:stefania.cacovich@cnrs.fr, giulia.grancini@unipv.it

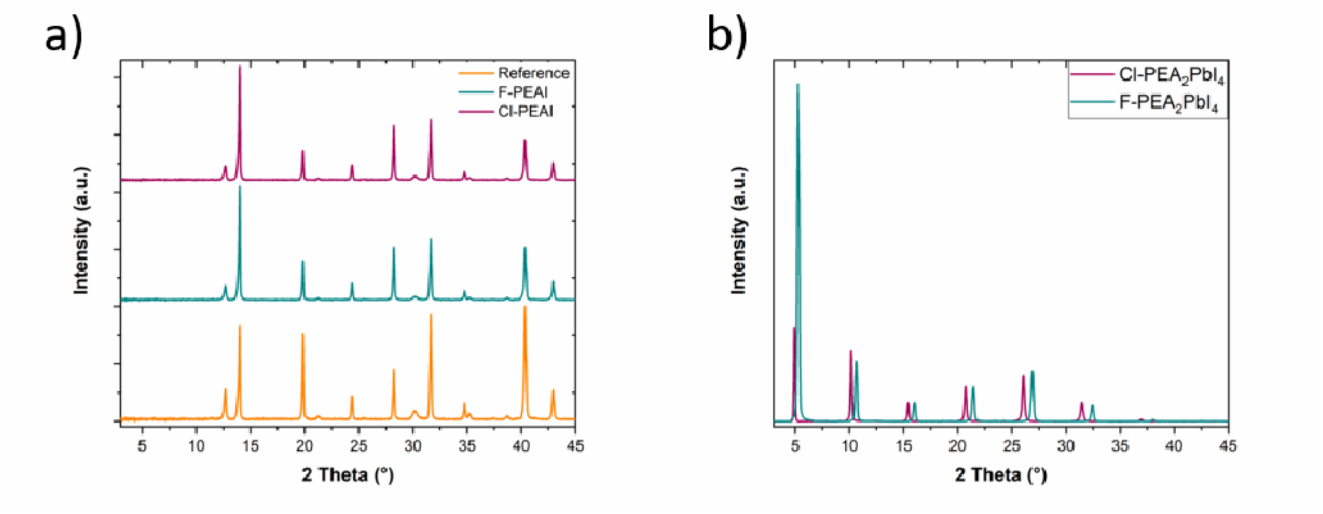

Supplementary Figure 1. a) XRD spectra of 3D perovskite thin films thin film deposited on ITO/PTAA/Cation substrates for reference and after Cl-PEAI and F-PEAI addition on top of the 3D and b) pure 2D perovskite Cl-PEA_2PbI_4 and F-PEA_2PbI_4

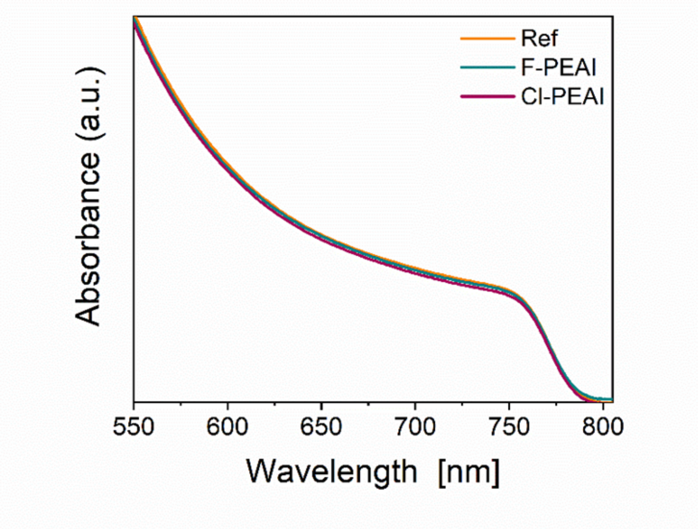

Supplementary Figure 2. Absorbance spectra of reference, Cl-PEAI and F-PEAI passivated thin films

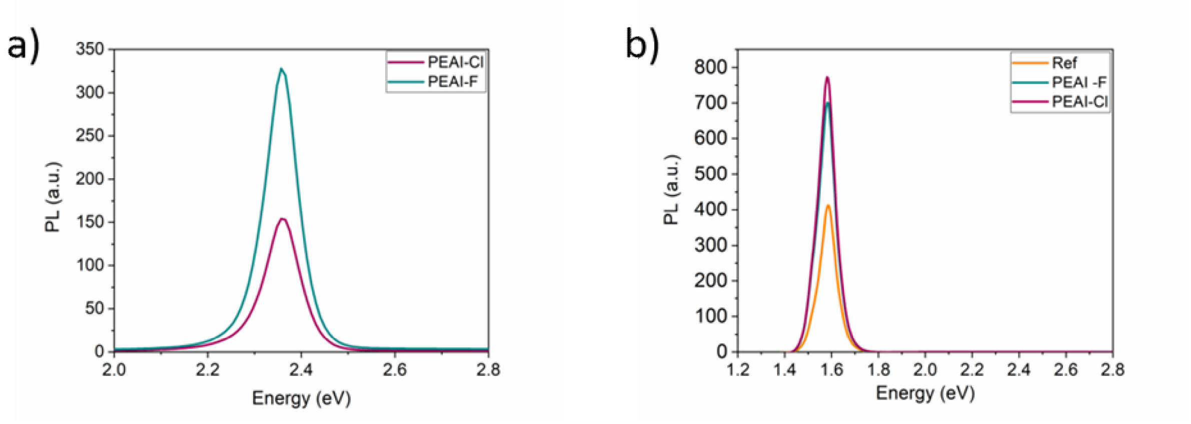

Supplementary Figure 3. a) PL spectra of 2D perovskite thin films showing an emission peak at approximately 2.35 eV. b) PL spectra for neat perovskite thin films deposited on glass. The spectra were recorder in the range going from 1.4 eV to 2.8 eV. No characteristic peak from 2D perovskite can be observed in the range 2.0 – 2.6 eV when F-PEAI and Cl-PEAI were added at the absorber interfaces.

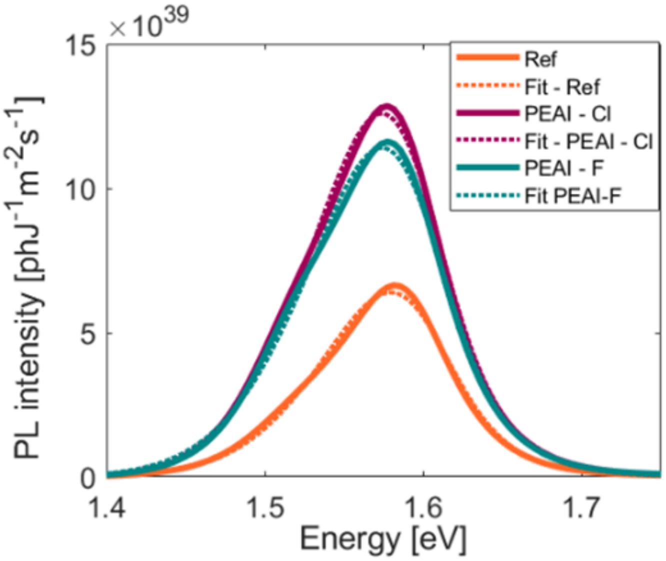

Supplementary Figure 4. Absolute calibrated PL spectra and relative fits of neat perovskite thin films deposited on glass.

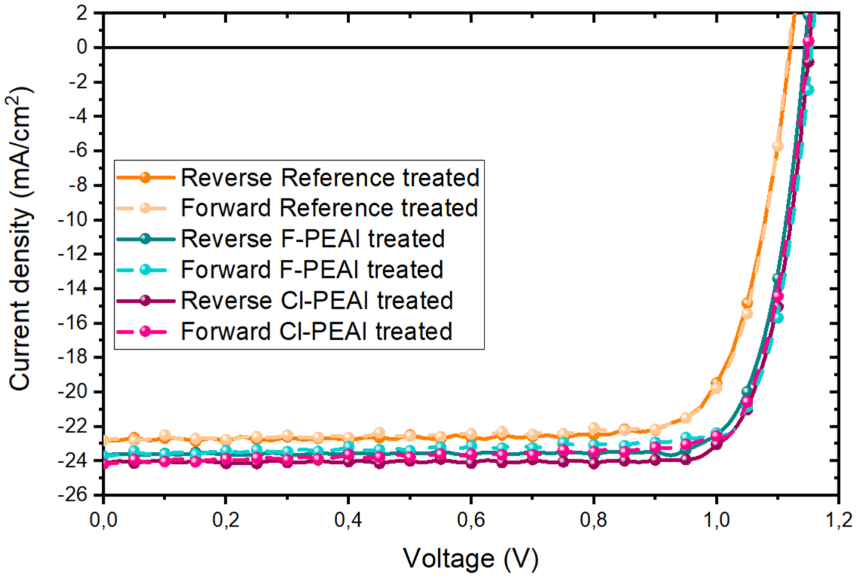

Supplementary Figure 5. J/V curve of the reference device and Cl-PEAI and F-PEAI passivated devices.

	Jsc (mA/cm^2)	Voc (V)	FF (%)	$FF_{pseudoJV}$ (%)	FF_{id} (%)	η(%)	$\eta_{pseudoJV}$ (%)	η_{id} (%)	$\Delta FF_{pseudoJV}$ (%)	ΔFF_{id} (%)	Rs ($Ohm*cm^2$)	Rsh (Ohm*cm2)
Reference	21.78	1.11	80.71	84.84	90.17	19.51	20.51	2179	4.13	9.46	0.608	3760
F-PEAI treated	23.42	1.17	83.23	86. 61	90.17	22.81	23.73	24.69	3.38	6.94	0.064	3200
Cl-PEAI treated	23.79	1.15	83.55	86.22	90.17	22.86	23.59	24.66	3.06	6.62	0.182	3600

Supplementary Table 1. PV parameters of the reference, F-PEAI treated and Cl-PEAI treated devices.

One could estimate an ideal FF for the perovskite with a band gap of E_g=1.57 to be up to 90.17%. Considering the diode equation model, it is also possible to estimate the series and shunt resistance. The results are reported in **Supplementary Table 1**. The details of the model used which we implemented can be find in Pica et al. [1].

Radiative limit

The $\Delta\mu^{\mathrm{rad}}$ is defined as:

$$\Delta\mu^{rad} = kT \cdot \ln\left(\frac{I_{ph}}{q\phi_{em}}\right)$$

where I_{ph} is the photocurrent due to the considered illumination and ϕ_{em} is the PL emission of the absorber in thermal equilibrium with its surrounding at 300 K and kT the thermal energy of the charge carriers.

In our analysis, we consider the photocurrent generated by a monochromatic radiation at 405 nm with a 1 sun-equivalent power density to match the experimental conditions used in the steady measurements and directly compare the $\Delta\mu^{\mathrm{rad}}$ with the $\Delta\mu$ experimentally measured on bare absorbers and stacks.

The PL emission ϕ_{em} corresponds to $\phi_{em} = \int A(E)\phi_{BB}^{300K}(E)dE$, where ϕ_{BB}^{300K} is the blackbody radiation at 300K and $A(E)$ is the absorptivity of the perovskite, extrapolated from absorption measurements reported in **Supplementary Figure 2**.

The radiative V_{oc} ($V_{\mathrm{oc}}^{\mathrm{rad}}$) was calculated from :

$$V_{oc}^{rad} = \frac{kT}{q} \cdot \ln\left(\frac{J_{sc}}{J_{0rad}}\right)$$

Where kT is the thermal energy of the charge carriers, q is the elementary charge, J_{sc} is the short-circuit current density of the solar cell and J_{0rad} is the radiative current. Specifically:

$$J_{0rad} = q\int EQE(E)\phi_{BB}^{300K}(E)dE$$

with EQE being the external quantum efficiency of the full devices. This calculation is illustrated in **Supplementary Figure 6**where the EQE of one of the samples is reported (orange curve) together with the blackbody radiation (black curve) at the temperature of the experiment. The radiation current of charges per unit of energy is the product of the two later (blue curve). The radiative recombination current corresponds to the area in light blue under the blue curve. Interestingly, a narrow wavelength (energy) range of the EQE is of interest for the calculation of J_{0rad}

In particular, the quantity J_{0rad} defines both the lower limit for the actual saturation density J_0 of a real solar cell and the upper limit for the open-circuit voltage V_{oc}^{rad}. Moreover, the V_{oc}^{rad} not only depends on the optical properties and radiative recombination such as the $\Delta\mu^{rad}$ but also on the quantum efficiency and thus on collection and injection of carriers. This arises from the fact that the spectral dependence of the φ_{BB} weights the different spectral contribution of the EQE differently. However, the resulting changes in terms of V_{oc}^{rad} will be minimal as the ratio J_{sc}/ J_{0rad} enters logarithmically into V_{oc}^{rad}.

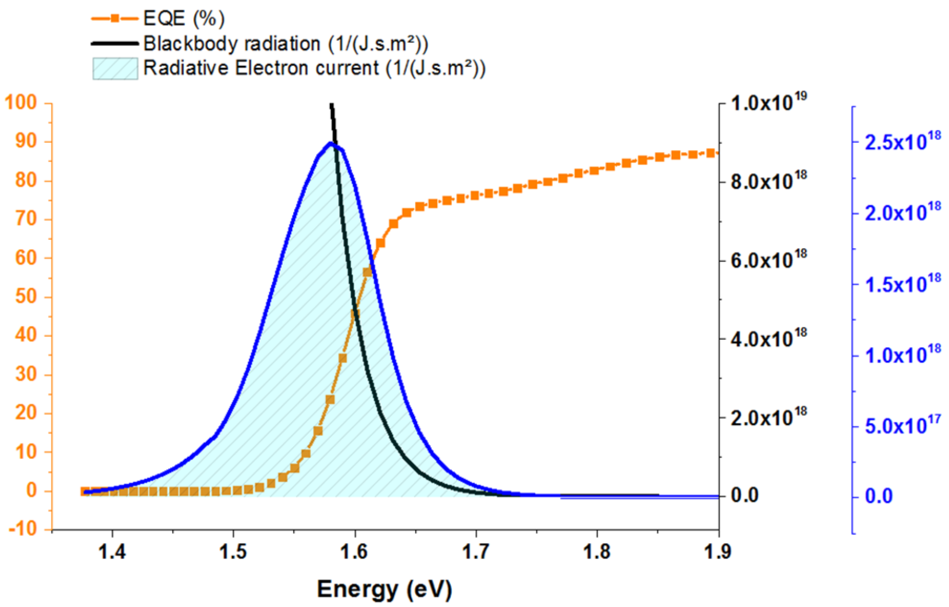

Supplementary Figure 6. External Quantum Efficiency of Cl-PEAI sample, Blackbody radiation and corresponding radiative current.

PL model for fitting steady-state measurements

The absolute photoluminescence data were fitted with the generalized Planck's law by:

$$I_{PL}(E) = \frac{\frac{2\pi}{h^3c^2}E^2 a(E)}{\exp\left(\frac{E-\Delta\mu}{kT}\right)}$$

Where h is the Planck's constant, c the speed of light, E the photon energy, a(E) the absorptivity of the sample taking into account sub-band gap absorption, Δμ the QFLS, and kT the thermal energy of the charge carriers.

The absorption is described by an ideal band-band term convoluted with a sub-bandgap absorption, in which the tail states are represented by an exponent factor θ. For a mono-exponential decay of the tail states in the gap, $\theta = 1$ and E_u corresponds to the Urbach energy[2].

$$a(E) = 1 - \exp\left(\frac{\alpha_0 d}{2E_u \Gamma\left(1+\frac{1}{\theta}\right)} \int_{-\infty}^{\infty} (exp\left(-\left|\frac{u}{E_u}\right|^{\theta}\right) \sqrt{E - E_g - udu}\right)$$

Fitted values for all the fitting parameters are reported in Table S1 (neat perovskite), Table S2 (half devices) and table S3 (full devices). In particular, we fixed three fitting parameters, namely the temperature T, the product α x d and the factor $\theta = 1$ in order to determine the Urbach Energy E_u (γ).

Sample	T (K)	QFLS (eV)	α x d (a.u.)	E_g (eV)	Θ (a.u.)	γ (eV)
**Ref**	293	1.24 ± 0.01	5	1.57 ± 0.01	1	0.017 ± 0.001
**Cl-PEAI**	293	1.25 ± 0.01	5	1.57 ± 0.01	1	0.018 ± 0.001
**F-PEAI**	293	1.25 ± 0.01	5	1.57 ± 0.01	1	0.017 ± 0.001

Supplementary Table 2. Fitting parameters for neat perovskite samples on average PL spectra**.**

Sample	T (K)	QFLS (eV)	α x d (a.u.)	E_g (eV)	Θ (a.u.)	γ (eV)
**Ref**	293	1.20 ± 0.01	5	1.57 ± 0.01	1	0.017 ± 0.001
**Cl-PEAI**	293	1.22 ± 0.01	5	1.57 ± 0.01	1	0.017 ± 0.001
**F-PEAI**	293	1.22 ± 0.01	5	1.57± 0.01	1	0.017 ± 0.001

Supplementary Table 3. Fitting parameters for half cells (HTL/perovskite) on average PL spectra**.**

Sample	T (K)	QFLS (eV)	α x d (a.u.)	E_g (eV)	Θ (a.u.)	γ (eV)
**Ref**	293	1.11 ± 0.01	5	1.56± 0.01	1	0.017 ± 0.001
**Cl-PEAI**	293	1.17 ± 0.01	5	1.57 ± 0.01	1	0.016 ± 0.001
**F-PEAI**	293	1.18 ± 0.01	5	1.57 ± 0.01	1	0.017 ± 0.001

Supplementary Table 4. Fitting parameters for full devices (HTL/perovskite/ETL) on average PL spectra.

Sample	Parameter	Median (eV)	Standard Deviation
**Ref**	QFLS	1.110	0.013
**Cl-PEAI**	QFLS	1.176	0.006
**F-PEAI**	QFLS	1.184	0.007
**Ref**	Eg	1.569	0.012
**Cl-PEAI**	Eg	1.572	0.006
**F-PEAI**	Eg	1.575	0.006
**Ref**	Eu	0.0167	0.002
**Cl-PEAI**	Eu	0.0164	0.003
**F-PEAI**	Eu	0.0162	0.003

Supplementary Table 5. Median and standard deviation values calculated for QFLS, E_g and E_u maps showed in Figure 3.

Model for TR-measurements

The model we used to interpret the decays is the 1d-Drift-Diffusion model. We implemented a simplified model both for full-cell devices and thin-films. This model is the following[4].

We consider a slab of intrinsic semiconductor of thickness L. The laser pulse will generate excited charges in the device. We consider that the electron and hole density to be equal at all position in the thickness and time. We model the time and space (in thickness) dependent photo generated carrier density $\Delta n(z,t)$ with the 1d-Drift-Diffusion equation:

$$\frac{\partial \Delta n}{\partial t} = D\frac{\partial^2 \Delta n}{\partial z^2} - k_1 \Delta n - k_2 \Delta n^2 + g(z,t) \tag{S1}$$

With the effective diffusion coefficient D, the SRH recombination constant k_1 and the radiative external recombination coefficient k_2. The laser pulse is modeled via the time dependent generation rate g via:

$$g(z,t) = \left[n_\gamma \alpha e^{-\alpha z}\right] \times \left[\frac{1}{\sqrt{2\pi}\sigma} \exp\left(\frac{-t^2}{2\sigma^2}\right)\right]$$

That is Beer-Lambert's absorption law with α the absorption coefficient at the laser wavelength $\alpha(532nm) = 1.3e5\ cm^{-1}$ and n_γ the fluence in ph.cm^{-2}pulse^{-1} which is varied during the experiment. The right part of g is a Gaussian temporal profile of duration $\sigma = 5ps$ in the simulation.

The boundary conditions to solve **Eq S1** are the following:

$$\begin{cases} \left.\frac{\partial \Delta n}{\partial z}\right|_{z=0} = S_{top}\Delta n(z=0,t) \\ \left.\frac{\partial \Delta n}{\partial z}\right|_{z=L} = -S_{bot}\Delta n(z=L,t) \end{cases}$$

They represent non radiative recombination at the interfaces.

The definition of the photoluminescence signal in this model is taken as:

$$I_{\mathrm{PL}}(t) = A\int_0^L dz\,\Delta n^2$$

With A a constant that represents geometrical factors as well as the radiative recombination coefficient k_2. Since the fitted decays are all normalized, the value of A has no effect on the model.

The implementation of the model is made via a Matlab code using the **pdepe** function to solve the partial differential equation. A fitting procedure was coded and has the following properties:

1. A unique model is fitted for all the selected fluences. That means that only one value for the physical parameters is fitted – no fluence dependent values are fitted.
2. A unique model is fitted for the experimental curves acquired in top or bottom illumination configuration. The model takes into account which curves comes from which experiment.
3. The uncertainties on the fitted parameters are estimated using the **nlparci** method. They are an estimate of the uncertainties – which are difficult to determine for this non-linear fitting method.

The reference thin film was used to fit the diffusion coefficient – which was then fixed to the value of $4.5e-3\ cm^2s^{-1}$ $(\pm 3\%)$ for all other fits, see **Supplementary Figure 7**. The fitting offers a very close correspondence with the experimental results. The correlations are relatively

low except for the correlation between the top surface and bulk non radiative recombination parameters S_{top} and k_1. This comes from the fact that both will have similar effects on the decays. This makes it impossible to distinguish independently bulk from top surface recombination in this framework. To test this hypothesis, we tried fitting the full cell devices with and without bulk recombination, see **Supplementary Figure 8**. The addition of bulk recombination was not found to improve the fitting result and no specific feature were modeled by this parameter. We therefore decided to neglect it for simplicity reasons and to attribute all non-radiative recombination to the interfaces.

Fitting the full cell devices gave us a radiative recombination coefficient higher than for the films on glass possibly due to a change in photon outcoupling. The fitted value for the full cells is: $k_2 = (2.5 \pm 1) \times 10^{-10}\ cm^3 s^{-1}$.

The three full cell devices were fitted using the procedure. The results are the following:

Parameter	Symbol	Value	Comment
Thickness	L	500nm	Not fitted
Bulk defect SRH coefficient	k_1	$0 s^{-1}$	Hypothesis to compare the impact of the surfaces
Radiative recombination coefficient	k_2	$(2.5 \pm 1) \times 10^{-10}\ cm^3 s^{-1}$	Fitted from the full cell reference
Diffusion coefficient	D	$4.5 \times 10^{-3}\ cm^2 s^{-1}$ ($\pm 3\%$)	From a fit of the thin film reference.

Supplementary Table 6. Model parameters values.

The corresponding figures are shown in **Supplementary Figure 10**. Even though the model is extremely simplified, it is able to describe correctly both the top and bottom illumination

configuration as well as the change in fluence with a unique model for each sample. The fitted recombination values show a passivation of the cells compatible with the other measurements.

A question is the role of the extracting layers on the TR-PL decay. Following the work of Krückemeier and colleagues[3], we think that the high fluence behavior of the full cell devices is close to the behavior of a single absorbing layer without charge transport layers. This is exemplified here by the fact that the simplest model is capable of qualitatively reproducing the observed trends of the TR-PL decays. We believe that the simplest model possible should be applied to all data sets in order to avoid overfitting. At high fluence and in open circuit condition, we deem that the effects of the charge transport layers are not the main drive of the decays.

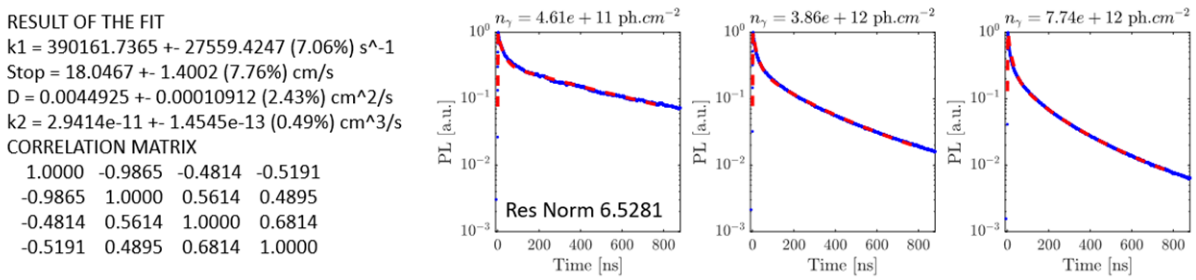

Supplementary Figure 7. TR-FLIM fit result on the thin film reference (no passivation layer added) perovskite, top illumination. The Bottom recombination velocity was set to zero. (Blue) Experimental result (Red) Fitted model (unique for the three fluences).

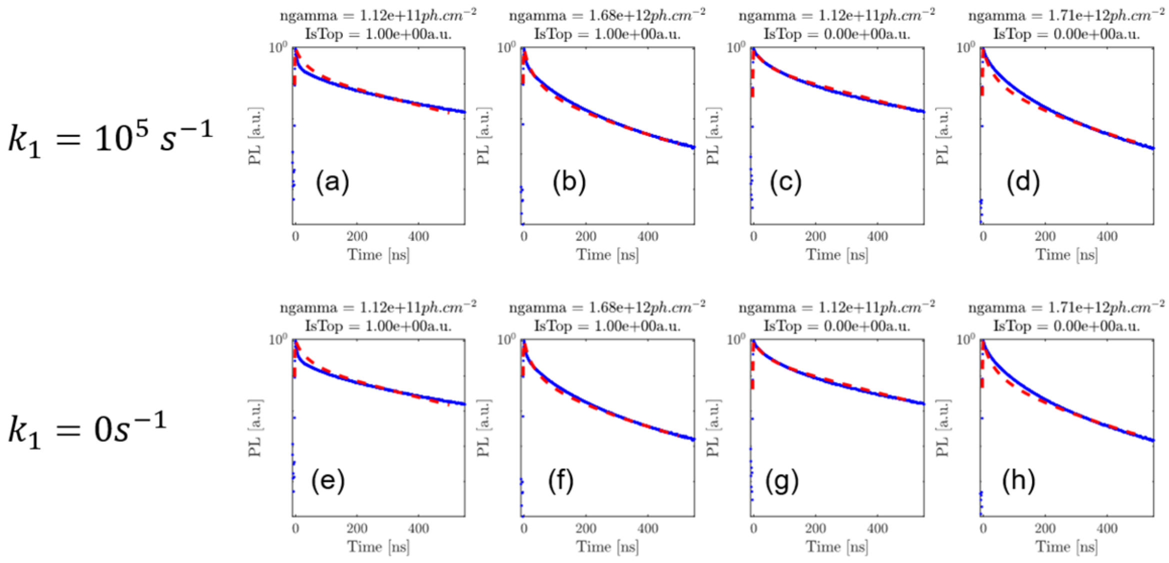

Supplementary Figure 8.TR-FLIM (spatially integrated) decays for the Cl-PEAI passivated full cell device. Effect of neglecting the bulk recombination on the fitting results. (First row) With bulk recombination fixed to 105 s-1. (a-b) Top Illumination (c-d) Bottom illumination. (Second row) Without bulk recombination. (e-f) Top illumination (g-h) Bottom illumination. The fitted values go from $S_{top} = 104\ (\pm 3\%) cm.s^{-1}$ with bulk recombination to $S_{top} = 110\ (\pm 3\%)\ cm.s^{-1}$ without bulk recombination. For the bottom recombination they go from $S_{bot} = 60\ (\pm 3\%)\ cm.s^{-1}$ to $S_{bot} = 64\ (\pm 3\%)\ cm.s^{-1}$.

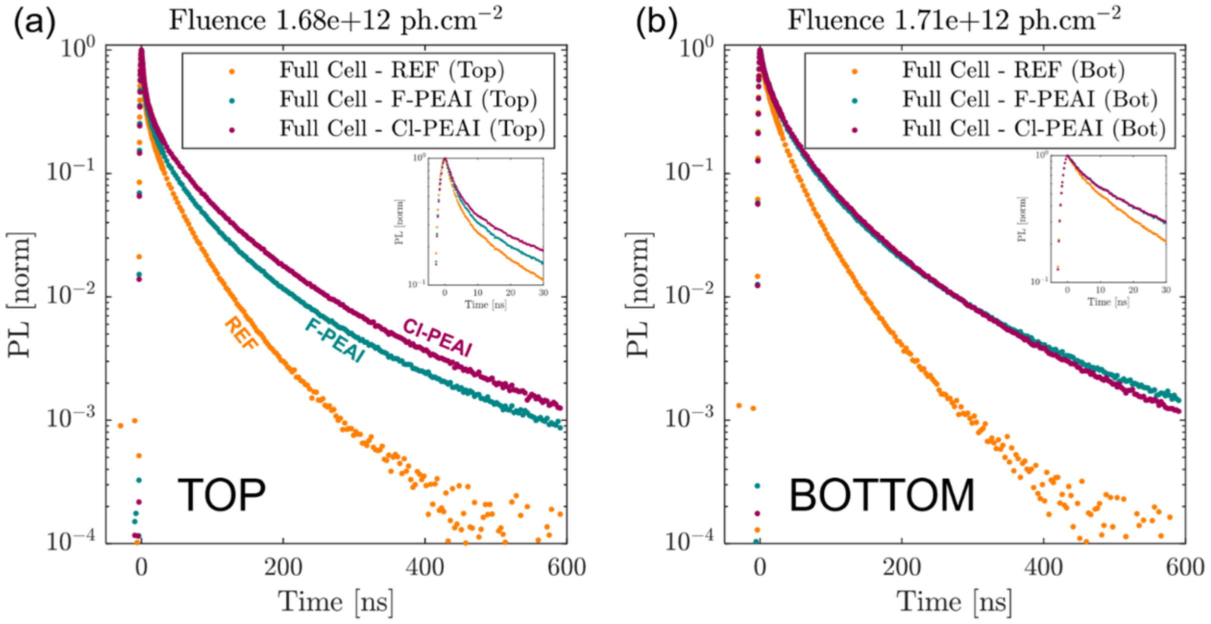

Supplementary Figure 9. TR-FLIM (spatially integrated) decays on full cell devices. High fluence. (a) Top illumination configuration. (b) Bottom illumination configuration.

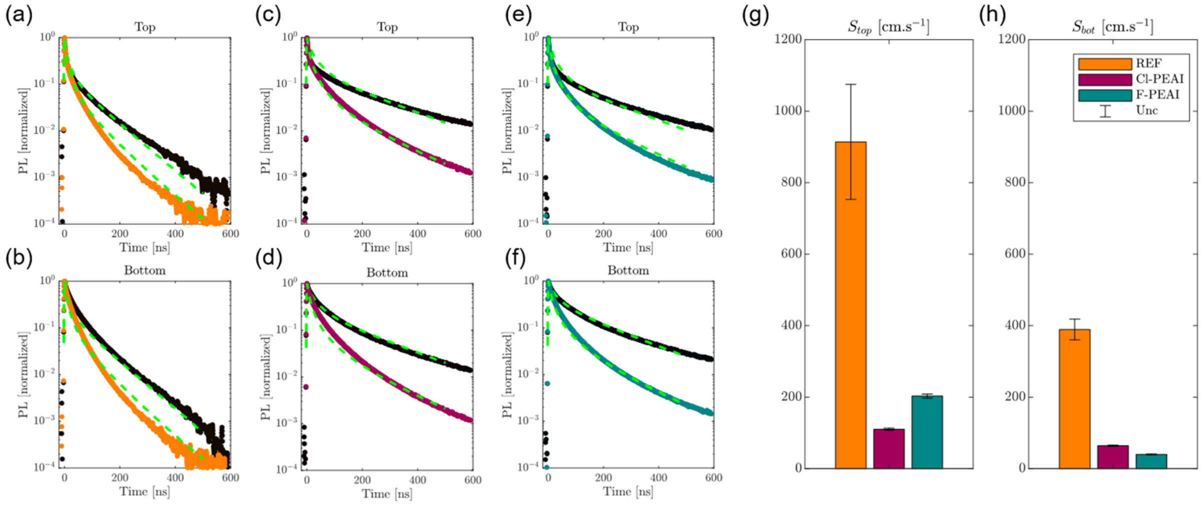

Supplementary Figure 10. Fitting result of the decays obtained on full-cells. Green dotted line, fitting model. Colored dots, experimental data. The dark curves is the low fluence one, while the bright one corresponds to high fluence. (a-b) Reference cell (with no passivation), top and bottom sides (c-d) Cell with added Cl-PEAI passivation (e-f) Cell with added F-PEAI passivation. For the 3 samples, only one model represents the whole dataset (2 fluences, 2 sides). (g) Fitted value of top surface recombination velocity (h) Fitted value of bottom surface recombination velocity.

Repartition of the recombination in the model

In this section we use our fitted model to attribute the decay to its origins. We can compute theoretically the time derivative of the PL signal as:

$$\frac{dI_{\mathrm{PL}}}{dt}(t) = -2A\left[\underbrace{k_1\int_0^L dz\,\Delta n^2}_{\text{(i)}} + \underbrace{S_{top}\Delta n^2(z=0,t)}_{\text{(ii)}} + \underbrace{S_{bot}\Delta n^2(z=0,t)}_{\text{(iii)}} + \underbrace{D\int_0^L dz\left(\frac{\partial \Delta n}{\partial z}(z,t)\right)^2}_{\text{(iv)}} + \underbrace{k_2\int_0^L dz\,\Delta n^3}_{\text{(v)}}\right]$$

This decomposition may also be found in the paper by Maiberg et al.[4]. Each of the terms inside the brackets can be attributed to a physical interpretation. Term (i) corresponds to the part of

the reduction of the PL signal due to SRH recombination. Term (ii) to the part of the PL decay due to top surface recombination. Term (iii) to bottom surface. Term (iv) is the contribution of diffusion to the decay. And term (v) is due to radiative recombination.

With this integral decomposition we can monitor the share of the total value of the PL as well as its physical origin as a function of time for modeled decays. We compute each of the integrals and normalize the obtain values by the numerically computed derivative of the PL signal. We obtain **Supplementary Figure 11**.

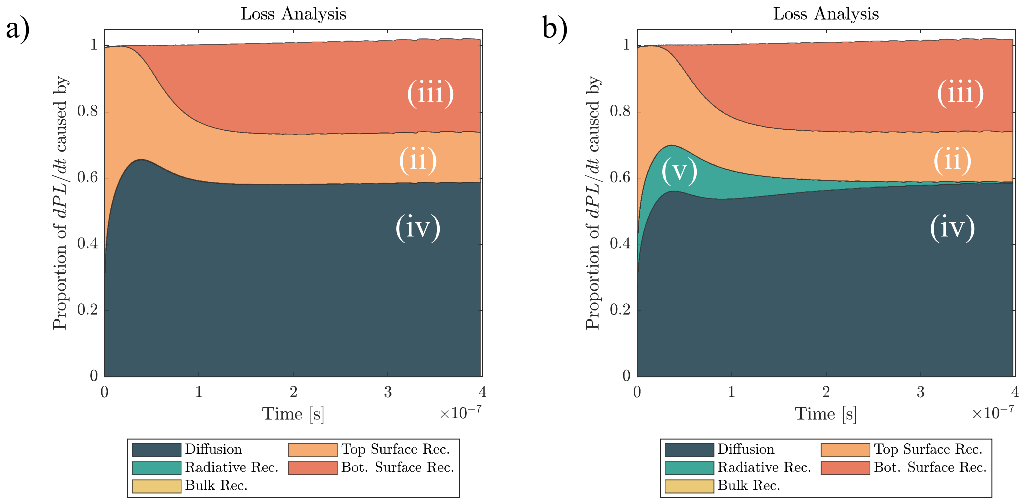

Supplementary Figure 11. Simulation of the decay causes for the fitted model of the full-cell reference. (a) ~10^{11} ph.cm^{-2} illumination (b) ~1.5 10^{12} ph.cm^{-2} illumination. Parameters are the following: D=4.5e-3 cm²/s ; k_2 = 2.5e-10 cm^{3}s^{-1}; S_{top} = 900 cm/s ; S_{bot} = 400 cm/s ; α= 1.3e5 cm^{-1}.

As explained in the section "Model for TR-PL experiment", we considered k_1=0 s^{-1} and therefore the term (i) related to SRH recombination is negligible. The initial fast decay of **Figure 2(d)** is caused mainly by top-surface non radiative recombination, see **Supplementary Figure 11(a)** zone (ii). In the case of high excitation (1.5 10^{12} ph/cm²/pulse), the radiative recombination also plays a role in the decay for times smaller than 200 ns, but their contribution in explaining the PL decay never surpasses 20%.

Computation of the decay time

In this section, we describe our reasoning to compute the decay time. Mainly, we tackle the question of computing the decay time when the charge carrier density in the device is close to the estimated 1 sun excitation density, ie 10^{15} cm^{-3}.

For an intrinsic material we have the following scaling: $PL \propto \Delta n^2$ or we can write it as $\Delta n \propto PL^{1/2}$. This implies that a loss of two orders of magnitude of PL leads to a loss of one order of magnitude of carrier density. We have two sets of acquisitions for each sample, one with an initial carrier density of 1.5 10^{17} cm^{-3} (high fluence, 150 suns) and one with an initial carrier density of 10^{11} cm^{-3} (low fluence, 10 suns).

For the high fluence acquisition, we would need to observe at least 4 orders of magnitude to reach 1 sun equivalent carrier density. This is not possible since the noise of our experimental setup is reached approximatively in this regime.

For the low fluence acquisition, we only need to wait for 2 orders of magnitude of PL decrease to reach a density of approximately 10^{15} cm^{-3} ie, 1 sun acquisition. For this acquisition, this is the case in the [300; 500] ns region, see **Figure 2(d).**

Once we have selected a time window, the decay time is computed by using the following model:

$$\log PL(t) = cte - \frac{t}{\tau} = a + bt$$

We fit a linear behavior for the logarithm of the PL in the corresponding temporal window. The decay time is defined as the inverse of the absolute value of the slope: $\tau = -1/b$.

To obtain the mapping of the decay time, we used our TR-FLIM acquisitions. One issue that arises is the noise at the level of each pixel. To be able to average out noise at the local level, we performed a temporal mean of the logarithm of decays of each pixel on different temporal windows noted $[t_i; t_{i+1}]$. Namely, we averaged the decays in the logarithm space and created a new dataset PL':

$$PL'\left(\frac{t_i + t_{i+1}}{2}\right) = \exp\left(\frac{1}{t_{i+1} - t_i}\int_{t_i}^{t_{i+1}} dt \log(PL(t))\right)$$

This is justified in the case of a mono-exponential decay, which we expect here after ~100ns after the pulse. We used the following time windows limits:

$$\{t_i\} = [90 \quad 140 \quad 190 \quad 290 \quad 490]\, ns$$

We obtained a set of 4 values of PL' for each pixel, from which we extracted the local decay time, as shown in **Figure 4**. For the low fluence acquisitions, the decay time was computed on the average decay over the images, provided in **Figure 2 (d)**. This is because local noise on the pixels prevented us from obtaining maps with a high enough signal to noise ratio.

References

1. Pica, G. *et al.* Accelerated Thermal Aging Effects on Carbon-Based Perovskite Solar Cells: A Joint Experimental and Theoretical Analysis. *Solar RRL* **5**, 2000759 (2021).
2. Katahara, J. K. & Hillhouse, H. W. Quasi-Fermi level splitting and sub-bandgap absorptivity from semiconductor photoluminescence. *Journal of Applied Physics* **116**, 173504 (2014).
3. Krückemeier, L., Krogmeier, B., Liu, Z., Rau, U. & Kirchartz, T. Understanding Transient Photoluminescence in Halide Perovskite Layer Stacks and Solar Cells. *Advanced Energy Materials* **11**, 2003489 (2021).
4. Maiberg, M. & Scheer, R. Theoretical study of time-resolved luminescence in semiconductors. II. Pulsed excitation. *Journal of Applied Physics* **116**, 123711 (2014).
