# Supplementary material for: Imaging and quantifying non-radiative losses at 23% efficient inverted perovskite solar cells interfaces
Source: Nat Commun. 2022 May 23;13:2868. doi: 10.1038/s41467-022-30426-0 (PMC9126963; doi:10.1038/s41467-022-30426-0)
Supplement: Supplementary file 2 — Solar Cells Reporting Summary [file 41467_2022_30426_MOESM2_ESM.pdf]

Corresponding author(s): Stefania Cacovich
DBPR NCOMMS-21-39093

Solar Cells Reporting Summary

Nature Research wishes to improve the reproducibility of the work that we publish. This form is intended for publication with all accepted papers reporting the characterization of photovoltaic devices and provides structure for consistency and transparency in reporting. Some list items might not apply to an individual manuscript, but all fields must be completed for clarity.

For further information on Nature Research policies, including our data availability policy, see Authors & Referees.

Experimental design

Please check: are the following details reported in the manuscript?

1. Dimensions

Item	Yes	No	Explanation
Area of the tested solar cells	☒	☐	Area of the cell reported in the Experimental Methods of the main manuscript
Method used to determine the device area	☒	☐	The area corresponds to the overlapping region among the ITO and top metal contact

2. Current-voltage characterization

Item	Yes	No	Explanation
Current density-voltage (J-V) plots in both forward and backward direction	☒	☐	Accurate description in the methods and see Figure S5 in the supplementary information
Voltage scan conditions<br>*For instance: scan direction, speed, dwell times*	☒	☐	Reverse-forward, step 0.025V, 0.1s dwell time. All the details in the Experimental Methods of the main manuscript
Test environment<br>*For instance: characterization temperature, in air or in glove box*	☒	☐	The environment conditions in the Experimental Methods of the main manuscript
Protocol for preconditioning of the device before its characterization	☐	☒	No preconditioning of device is used
Stability of the J-V characteristic<br>*Verified with time evolution of the maximum power point or with the photocurrent at maximum power point; see ref. 7 for details.*	☐	☒	*Explain why this information is not reported/not relevant.*

3. Hysteresis or any other unusual behaviour

Item	Yes	No	Explanation
Description of the unusual behaviour observed during the characterization	☐	☒	Unusual behaviour or hysteresis are not present in the JV curves see Figure S5 in the supplementary information
Related experimental data	☒	☐	JV curves without hysteresis are reported in Figure S5 in the supplementary information

4. Efficiency

Item	Yes	No	Explanation
External quantum efficiency (EQE) or incident photons to current efficiency (IPCE)	☐	☒	*Explain why this information is not reported/not relevant.*
A comparison between the integrated response under the standard reference spectrum and the response measure under the simulator	☐	☒	*Explain why this information is not reported/not relevant.*
For tandem solar cells, the bias illumination and bias voltage used for each subcell	☐	☒	No tandem cells are present in this paper

5. Calibration

Item	Yes	No	Explanation
Light source and reference cell or sensor used for the characterization	☒	☐	The Light source and the reference cell are reported in the Experimental Methods of the main manuscript
Confirmation that the reference cell was calibrated and certified	☒	☐	The reference cell was still in the guarantee period

Calculation of spectral mismatch between the reference cell and the devices under test

- [x] Yes
- [ ] No

Spectral Mismatch is reported in the Experimental Methods of the main manuscript

6. Mask/aperture

Size of the mask/aperture used during testing

- [ ] Yes
- [x] No

no shadow mask is used

Variation of the measured short-circuit current density with the mask/aperture area

- [ ] Yes
- [x] No

Explain why this information is not reported/not relevant.

7. Performance certification

Identity of the independent certification laboratory that confirmed the photovoltaic performance

- [ ] Yes
- [x] No

No test of indipendent certification laboratory is performed in this paper

A copy of any certificate(s)
Provide in Supplementary Information

- [ ] Yes
- [x] No

not applicable

8. Statistics

Number of solar cells tested

- [x] Yes
- [ ] No

The dots in the box chart Figure 1 in the main manuscript is equal to the number of the solar cells tested

Statistical analysis of the device performance

- [x] Yes
- [ ] No

The statistics are present in the box chart Figure 1 in the main manuscript is equal to the number of the solar cells tested

9. Long-term stability analysis

Type of analysis, bias conditions and environmental conditions
For instance: illumination type, temperature, atmosphere humidity, encapsulation method, preconditioning temperature

- [ ] Yes
- [x] No

In this paper it is not present long-term stability tests
